# Western blot analysis of BK channel *β*1‐subunit expression should be interpreted cautiously when using commercially available antibodies

**DOI:** 10.14814/phy2.12189

**Published:** 2014-10-29

**Authors:** Yogesh Bhattarai, Roxanne Fernandes, Mark M. Kadrofske, Lizbeth R. Lockwood, James J. Galligan, Hui Xu

**Affiliations:** 1 Neuroscience Program, Michigan State University, East Lansing, Michigan; 2 Department of Pharmacology and Toxicology, Michigan State University, East Lansing, Michigan; 3 Department of Pediatrics and Human Development, Michigan State University, East Lansing, Michigan

**Keywords:** Anti‐BK channel *β*1‐subunit antibody, BK *β*1‐subunit expression, BK *β*1‐subunit knockout, sensitivity and specificity, smooth muscle cell

## Abstract

Large conductance Ca^2+^‐activated K^+^ (BK) channels consist of pore‐forming *α*‐ and accessory *β*‐subunits. There are four *β*‐subunit subtypes (*β*1–*β*4), BK *β*1‐subunit is specific for smooth muscle cells (SMC). Reduced BK *β*1‐subunit expression is associated with SMC dysfunction in animal models of human disease, because downregulation of BK *β*1‐subunit reduces channel activity and increases SMC contractility. Several anti‐BK *β*1‐subunit antibodies are commercially available; however, the specificity of most antibodies has not been tested or confirmed in the tissues from BK *β*1‐subunit knockout (KO) mice. In this study, we tested the specificity and sensitivity of six commercially available antibodies from five manufacturers. We performed western blot analysis on BK *β*1‐subunit enriched tissues (mesenteric arteries and colons) and non‐SM tissue (cortex of kidney) from wild‐type (WT) and BK *β*1‐KO mice. We found that antibodies either detected protein bands of the appropriate molecular weight in tissues from both WT and BK *β*1‐KO mice or failed to detect protein bands at the appropriate molecular weight in tissues from WT mice, suggesting that these antibodies may lack specificity for the BK *β*1‐subunit. The absence of BK *β*1‐subunit mRNA expression in arteries, colons, and kidneys from BK *β*1‐KO mice was confirmed by RT‐PCR analysis. We conclude that these commercially available antibodies might not be reliable tools for studying BK *β*1‐subunit expression in murine tissues under the denaturing conditions that we have used. Data obtained using commercially available antibodies should be interpreted cautiously. Our studies underscore the importance of proper negative controls in western blot analyses.

## Abstract (precis)

Commercially available anti‐BK β1‐subunit antibodies either detected protein bands of the appropriate molecular weight in tissues from both WT and BK β1‐KO mice or failed to detect protein bands at the appropriate molecular weight in tissues from WT mice. These commercially available antibodies are not reliable tools for studying BK β1‐subunit expression in murine tissues. Data obtained using these antibodies should be interpreted cautiously.

## Introduction

BK (large conductance Ca^2+^‐activated K^+^) channels are expressed in many mammalian cell types and BK channel activity is an important regulator of cell excitability. BK channels are composed of pore‐forming *α*‐subunits and accessory *β*‐subunits that modulate *α*‐subunit Ca^2+^ sensitivity and channel activity (Nelson et al. 1995; Brenner et al. 2000). There are four subtypes (*β*1–*β*4) of BK channel *β*‐subunits. The BK *β*1‐subunit is largely smooth muscle cell (SMC) specific, although it has been found in the distal nephron of murine kidney (Grimm et al. 2007), BK *β*2‐ and *β*3‐subunits are expressed in endocrine cells (Xia et al. 1999; Braun et al. 2008), while BK *β*4‐subunits are expressed in neurons (Meera et al. 2000) and in the distal nephron of the kidney (Grimm et al. 2007). In SMCs, BK channels stabilize membrane potential and excitability through negative feedback modulation. Activation of BK channels causes SMC hyperpolarization, L‐type Ca^2+^ channel closure, and SMC relaxation (Nelson et al. 1995). Downregulation of BK *β*1‐subunit expression may contribute to human diseases including metabolic disorders (Zhang et al. 2010), hypertension (Yang et al. 2013), heart failure (Wan et al. 2013), asthma (Semenov et al. 2011), maladaption of uteroplacental circulation (Hu et al. 2012), urinary bladder overactivity (Petkov et al. 2001), and gastrointestinal motility disorders (France et al. 2012). Downregulation of BK *β*1‐subunit expression reduces *α*‐subunit Ca^2+^ sensitivity, therefore, causing SMC membrane depolarization and increased muscle contractility (Brenner et al. 2000; Plüger et al. 2000; Xu et al. 2011). BK *β*1‐subunit expression in pathological situations is mainly affected by signaling at the post‐transcriptional level where protein synthesis is reduced (Nelson et al. 1995; Grimm et al. 2009; Xu et al. 2011; Shi et al. 2013a) or protein degradation is accelerated (Zhang et al. 2010; Lu et al. 2012). Therefore, accurate measurement of BK *β*1‐subunit protein expression is crucial in studies of BK channel dysfunction in diseases.

The anti‐BK *β*1‐subunit antibodies used in published studies are either custom made (Lu et al. 2012; Yi et al. 2014) or purchased commercially. BK *β*1‐subunit protein expression is typically measured using western blot analysis. Importantly, the specificity and sensitivity of commercially available BK *β*1‐subunit antibodies have not been tested or confirmed in tissues from BK *β*1‐subunit knockout (KO) mice, although BK *β*1‐KO mice have been generated by two research groups (Brenner et al. 2000; Plüger et al. 2000). Recently, the specificity of commercially available antibodies has been questioned if antibody reactivity is not carefully characterized by using proper controls – for example, testing the antibody in tissues from knockout mice (Saper 2005). BK *β*1‐KO mice have been widely used as an animal model to study the contribution of BK channel dysfunction in hypertension (Grimm et al. 2007; Xu et al. 2011), kidney dysfunction (Grimm et al. 2007, 2009) septic shock (Xu et al. 2012, 2014), heart failure (Wan et al. 2013), asthma (Evseev et al. 2013), metabolic disorders (Lynch et al. 2013), irritable bowel syndrome (France et al. 2012), and urinary bladder overactivity (Petkov et al. 2001). These studies have demonstrated the functional deficiencies of BK channel in tissues from BK *β*1‐KO mice. Conclusions about the physiological significance of BK *β*1‐subunits in health and disease have been based, in part, on studies done in mice.

We used western blot analysis to test the specificity and sensitivity of six commercially available BK *β*1‐subunit antibodies from five manufacturers in BK *β*1‐subunit enriched SM tissues (mesenteric arteries, MA, and colons) and non‐SM tissues (cortex of kidney) from wild‐type (WT) and BK *β*1‐KO mice (Brenner et al. 2000; Grimm et al. 2007). We confirmed that BK *β*1‐KO mice lack *β*1‐subunit mRNA using RT‐PCR analysis.

## Materials and Methods

### Animals

Homozygous breeder male and female BK *β*1‐KO mice were a gift from Dr. Robert Brenner (University of Texas Health Science Center, San Antonio). BK *β*1‐KO mice are congenic as a result of seven generations of inbreeding to the C57BL/6 line and maintained originally as homozygous lines at Michigan State University (Xu et al. 2011, 2012, 2014; France et al. 2012). Pups of BK *β*1‐KO mice were weaned at 3 weeks. Age‐matched WT (C57BL/6) mice were purchased from Jackson Laboratories (Bar Harbor, ME). All mice were fed a normal diet. Mice used in our studies were at 20–24 weeks of age (male). All studies were conducted in accordance with the National Institutes of Health Guide for the Care and Use of Laboratory Animals (NIH Publication No. 85–23, revised 1996) and approved by the Michigan State University Institutional Animal Care and Use Committee.

### Western blot

Mesenteric arteries, colons, and cortex of kidney from WT and BK *β*1‐KO mice were isolated in cold Krebs solution and stored in −80°C until ready to be processed. Tissue was homogenized with a mortar and pestle that had been precooled with liquid nitrogen. Tissues were lysed with 1× lysis buffer (62.5 mol/L Tris HCl pH 6.8, 2% SDS, 10% Glycerol,) containing protease inhibitor cocktail (2 *μ*L/100 *μ*L lysis buffer) (P8340; Sigma‐Aldrich, St. Louis, MO), 10 mmol/L sodium fluoride (S7920; Sigma‐Aldrich), and 1 mmol/L sodium orthovanadate (S6508; Sigma‐Aldrich). Protein levels were quantified with Sigma's Bicinchoninic Assay (BCA1 and B9643; Sigma‐Aldrich). Samples were mixed 1:1 with 2× sample buffer (62.5 mmol/L Tris HCl pH 6.8, 2% SDS, 25% (w/v) glycerol, 0.01% bromophenol blue) (161‐0737; Bio‐Rad, Hercules, CA) containing 0.713 mol/L 2‐mercaptoethanol (M6250; Sigma‐Aldrich) and boiled for 5 min at 95–100°C. Protein (30–100 *μ*g for MA and colons, 500 *μ*g for kidney) were loaded on SDS‐PAGE (10–12%) gel along with the full‐range rainbow molecular weight marker (RPN800E; GE Healthcare, Pittsburgh, PA); separated at 100–120 V in running buffer (50 mmol/L Tris base, 384 mmol/L glycine, 0.1% SDS), and electrotransferred in transfer buffer (25 mmol/L Tris base, 192 mmol/L glycine, 20% methanol, overnight at 30 V (or at 100 V for 1 h) onto Amersham Hybond ECL Nitrocellulose Membrane (RPN68D; GE Healthare) or PVDF membrane (IPVH00010; EMD Millipore, Billerica, MA). Ponceau S was used to verify efficient protein transfer and to verify equal amounts of total protein loaded per lane. Samples were blocked for 1 h at room temperature in 5% milk‐TBS‐T, 5% BSA‐TBS‐T, or 4% chicken ovalbumin‐TBS‐T.

We tested six commercially available BK *β*1‐subunit antibodies from five manufacturers (Table 1). We also tested an anti‐BK *α*‐subunit antibody (APC‐107, anti‐*KCa1.1*, 1:500; Alomone Labs, Jerusalem, Israel) in protein extracts from colons and kidneys, to confirm BK channel expression in these tissues. Protein loading and transfer were confirmed by reblotting for *β*‐actin (A2228, anti‐*β*‐Actin, 1:1000; Sigma‐Aldrich) on each membrane after the BK *β*1‐subunit antibody was stripped. Membranes were blotted overnight at 4°C or 2 h at room temperature, with anti‐BK *α*‐ or *β*1‐subunit primary antibodies used at the manufacturers' suggested dilutions and after primary antibody incubation with antigen peptide (1:100) overnight (Table 1). The following day membranes were washed 3 × 10 min intervals with TBS‐T, and blotted with the appropriate HRP‐conjugated secondary antibodies (Table 1) for 1 h at room temperature in blocking solution. Membranes were then washed with TBS‐T for 3 × 10 min. Blots were imaged with Super Signal West Dura Extended Duration Substrate (34076; Thermo Scientific‐Pierce, Rockford, IL) using a LICOR‐FC imager, or using the Enhanced Chemiluminescence (ECL) Plus Western Blotting Detection System (RPN2133, GE Healthcare).

**Table 1.** List of commercially available anti‐BK *β*1‐subunit antibodies used in current studies

Manufacturer	Catalog #	Immunogen	MW (kDa)	Reactivity	Concentration	Secondary Ab
Alomone	APC‐036	2–17	28	H, R, M	1:100–1000	Anti‐Rabbit 1:2000 (sc‐2313; 7074S)
Abcam	Ab3587	90–103	35	H, R, M	1:500	Anti‐Rabbit 1:2000 (#7074S)
Pierce	PA1‐924	90–103	28	H, R, M	1:100‐1000	Anti‐Rabbit 1:2000 (sc‐2313; 7074S)
Pierce	PA5‐28284	1–191	28	H, M	1:100–1000	Anti‐Rabbit 1:2000 (sc‐2313)
Santa Cruz	sc‐14749	1–191	28	H, R, M	1:200	Anti‐Goat 1:500 (sc‐2056)
Biorbyt	orb‐101774	10–80	21	H, R, M	1:100–500	Anti‐Rabbit 1:2000 (sc‐2313; 7074S)

MW, manufacturer‐recommended molecular weight; H, human; R, rat; M, mouse; sc‐1213, donkey anti‐rabbit IgG‐conjugated to horseradish peroxidase, Santa Cruz Biotechnology; sc‐2056, donkey anti‐goat IgG‐conjugated to horseradish peroxidase, Santa Cruz Biotechnology; 7074S, goat anti‐rabbit IgG‐conjugated to horseradish peroxidase, Cell Signaling.

### Real‐time RT‐PCR

Mesenteric arteries, colons, and cortex of kidney were collected and stored in All Protect Reagent (76405; Qiagen, Valencia, CA) until ready to be processed. Total RNA was isolated with the MELT Total Nucleic Acid Isolation System kit (AM1983; Ambion, Carlsbad, CA) following the manufacturer's instructions. RNA isolates were reverse transcribed using High Capacity RNA to cDNA kits (4387406; Applied Biosystems, Carlsbad, CA), following the manufacturer's protocols. As a control for genomic DNA contamination, all cDNA synthesis reactions were set up with additional samples lacking reverse transcriptase. Resultant cDNA was used for real‐time PCR assays. Reactions (20 *μ*L) were prepared with TaqMan Fast Advanced master mix (4444556, Applied Biosystems) and inventoried with TaqMan Gene Expression assays (Mm00466621_m1 for KCNMB1, Rn01775763_g1 for Gapdh, Applied Biosystems). Samples were analyzed in duplicate. Real‐time RT‐PCR products at 40 cycles were also determined by agarose gel analysis (1.5% TBE agarose gel at 100 V for 1 h).

## Results

### Western blots in MA, colons, and kidneys from WT and BK β1‐KO mice

**Figure 1.**
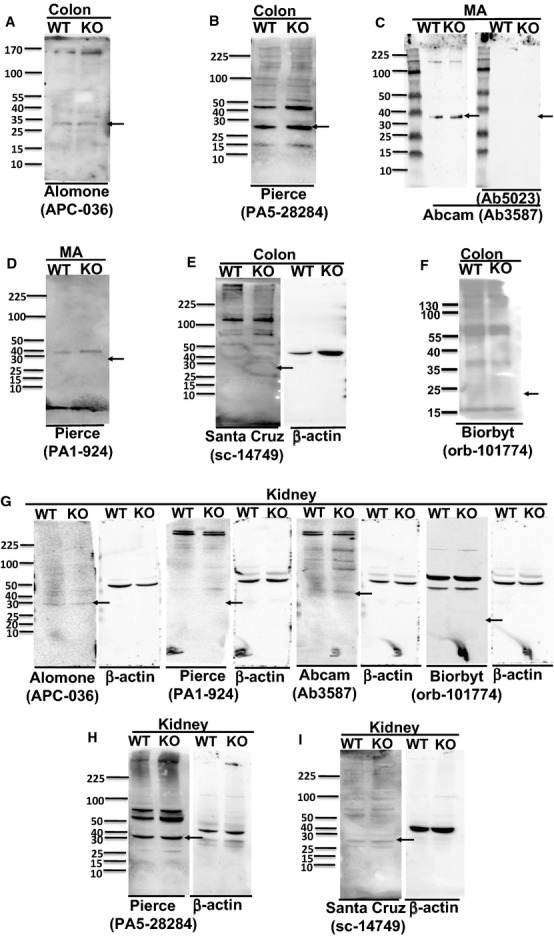
Representative western blot obtained using anti‐BK *β*1‐subunit antibodies in MA, colonic, or kidney tissues from WT and BK *β*1‐KO mice, (A) Alomone Labs (APC‐036), (B) Pierce (PA5‐28284), and (C) Abcam (Ab3587) antibodies detected a protein band at ~28 kDa or ~38 kDa in colons or MA from both mice. The bands in Abcam sets were diminished after preincubation of the primary antibody with the competing peptide. (D) Pierce (PA1‐924), (E) Santa Cruz (sc‐14749), and (F) Biorbyt (orb‐101774) antibodies did not detect any band at ~28 kDa or ~21 kDa in MA or colons from WT mice. (G) Alomone Labs (APC‐036), Pierce (PA1‐924), Abcam (Ab3587), and Biorbyt (Orb‐101774) antibodies did not detect protein band at ~28 kDa, ~38 kDa, or ~21 kDa in kidneys from WT mice. (H) Pierce (PA5‐28284) and (I) Santa Cruz (sc‐14749) antibodies detected the protein band at ~28 kDa in kidneys from both mice. *β*‐actin was reblotted on each membrane after anti‐BK *β*1‐subunit antibody was stripped. All representative blot images from kidney are in the tissue from same WT or BK *β*1‐KO mouse, and blotted with primary anti‐BK *β*1‐subunit antibody at 1:200. Arrows indicate the manufacturer's recommended molecular weight of BK *β*1‐subunit protein.

In SM tissues, APC‐036 (Alomone Labs) and PA5‐28284 (Thermo Scientific‐Pierce) anti‐BK *β*1‐subunit antibodies detected protein bands at ~28 kDa (manufacturer suggested molecular weight of BK *β*1‐subunit protein) in MA and colonic tissues from WT and BK *β*1‐KO mice, (Fig 1A, 1B). Ab3587 (Abcam, Cambridge, MA) anti‐BK *β*1‐subunit antibody detected a protein band at ~38 kDa (manufacturer's suggested molecular weight of BK *β*1‐subunit protein) in MA and colons from WT and BK *β*1‐KO mice (Fig 1C). Signals were blocked after preincubation of the Ab3587 antibody with its competing peptide (Ab5023) (Fig 1C). PA1‐924 (Thermo Scientific‐Pierce) anti‐BK *β*1‐subunit antibodies detected a protein band at ~40 kDa in MA and colons from WT and BK *β*1‐KO mice, but not at ~28 kDa (manufacturer suggested molecular weight of BK *β*1‐subunit protein) (Fig 1D). The sc‐14749 (Santa Cruz Biotechnology, Dallas, TX) and Orb‐101774 (Biorbyt, San Francisco, CA) antibodies detected multiple protein bands in MA and colonic tissues from WT and BK *β*1‐KO mice. However, none of these protein bands corresponded to a protein with a molecular weight of ~28 kDa or ~21 kDa (the manufacturer's suggested molecular weight of BK *β*1‐subunit protein) in tissues from WT mice, even when gels were loaded with 80 *μ*g of protein and incubated with high concentration of primary antibody (1:100) (Fig 1E, 1F). Protein loading and transfer were confirmed by *β*‐actin immune blot (Fig 1E). We repeated sc‐14749 and Orb‐101774 immunoblotting using a variety of protocols (See Methods). Results obtained using modified approaches were similar to the results described above. Tissues from six to eight animals were tested in each group.

In kidney tissues, APC‐036 (Alomone Labs), PA1‐924 (Thermo Scientific‐Pierce), and Ab3587 (Abcam) antibodies detected several faint protein bands in kidneys from WT and BK *β*1‐KO mice (Fig 1G). None of these protein bands corresponded to a protein with the manufacturer's suggested molecular weight of BK *β*1‐subunit protein in tissues from WT mice *only* (Fig 1G). The Orb‐101774 (Biorbyt) antibody detected two distinct protein bands in tissues from WT and BK *β*1 subunit KO mice, but failed to detect a band at ~21 kDa in tissues from WT mice (Fig 1G). PA5‐28284 (Thermo Scientific‐Pierce) and sc‐14749 (Santa Cruz Biotechnology) detected protein bands at the manufacturer's suggested molecular weight of BK *β*1‐subunit protein, but the bands were identical in tissues from both WT and BK *β*1‐KO mice (Fig 1H, 1I). Protein loading and transfer in each membrane were confirmed by *β*‐actin immune blots. Tissues from two animals were tested in each group.

### BK β1‐subunit mRNA expression in tissues from BK β1‐KO mice

**Figure 2.**
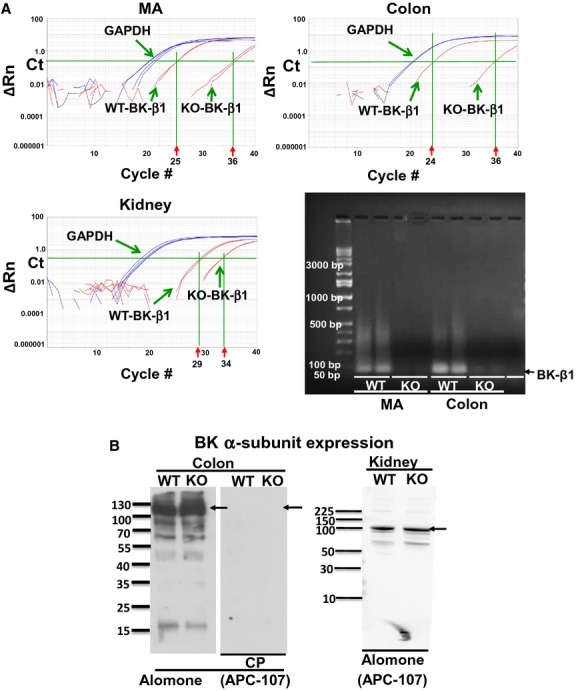
(A) Representative amplification plots and agarose gel separation of real‐time RT‐PCR analysis of BK *β*1‐subunit and GAPDH in MA, colons, and kidneys from WT and BK *β*1‐KO mice. The expression threshold was set at 0.22, a level above background fluorescence but within the linear phase of the amplification plot. The intersection between the threshold level and the amplification plot is the Ct value, which correlates with the amount of template in the sample. Ct values over 35 are excluded, as these values approach the sensitivity limits of the Taqman assay. Amplification of real‐time RT‐PCR products was seen at 75 bp in tissues from WT animals only. Con, nontemplate control. (B) Representative western blot obtained using anti‐BK *α*‐subunit antibody in colonic and kidney tissues from WT and BK *β*1‐KO mice. Antibody detected a protein band at ~100 kDa in all tissues from WT and BK *β*1‐KO mice. The signals are blocked by preincubation with the antibody competing peptide (CP). Arrows indicate the manufacturer's recommended molecular weight of BK *α*‐subunit protein.

To confirm the BK *β*1‐subunit gene has been deleted in BK *β*1‐KO mice, we measured expression of BK *β*1‐subunit mRNA levels in MA, colons, and cortex of kidneys from WT and BK *β*1‐KO mice using real‐time RT‐PCR. After 40 PCR cycles, the Ct values of BK *β*1‐subunit mRNA levels in MA, colons, and kidneys from WT mice were ~25, ~24, and ~29, respectively (Fig 2A,) but BK *β*1‐subunit mRNA was undetectable in tissues from BK *β*1‐KO mice (Fig 2A). Tissues from six to eight animals were tested in each group.

### Expression of BK α‐subunit in colons and kidneys

APC‐107 anti‐BK *α*‐subunit detected a protein band at ~100 kDa (manufacturer suggested molecular weight) in colonic and kidney tissues from WT and BK *β*1‐KO mice. Western blot signals were blocked after preincubation of the APC‐107 antibody with its competing peptide (Fig 2B).

## Discussion

We tested the specificity and sensitivity of commercially available BK *β*1‐subunit antibodies using tissues from WT and BK *β*1‐KO mice, and found that under denaturing conditions these antibodies lacked either specificity or sensitivity for BK *β*1‐subunit in BK *β*1‐subunit enriched tissues from C57BL/6 mice. The antibodies evaluated in this study have been used previously in different tissues including blood vessels, tracheal smooth muscle cells, and kidney from rats, mice, and humans (Matharoo‐Ball et al. 2003; Chang et al. 2006; Grimm et al. 2007, 2009; Yang et al. 2009, 2013; Albarwani et al. 2010; Xie et al. 2010; Zhang et al. 2010; Howitt et al. 2011; Ahn et al. 2012; Loot et al. 2012; Lu et al. 2012; Evseev et al. 2013; Kunduri et al. 2013; Shi et al. 2013a,b; Zheng et al. 2013; Evanson et al. 2014; Leo et al. 2014; Nystoriak et al. 2014; Pabbidi et al. 2014; Yi et al. 2014). However, our study is the first to test the specificity and sensitivity of all commercially available antibodies for detection of the BK *β*1‐subunit using BK *β*1‐subunit KO mice test.

The BK *β*1‐KO mice used in our study were generated using a viral vector (pPNT) to completely delete exon 2 of gene 27, which also includes a transcriptional terminator after the lacZ gene to prevent downstream expression of *β*1‐subunits (Brenner et al. 2000). Absence of BK *β*1‐subunit expression in the BK *β*1‐KO mice has been confirmed by RT‐PCR previously (Brenner et al. 2000) and in our study. BK *β*1‐subunit‐specific antibodies should identify a protein band with a molecular weight of 21–35 kDa (depending on the supplier's recommended molecular weight) in BK *β*1‐subunit enriched tissues obtained from WT mice. BK *β*1‐subunit contains 191 amino acids, calculated molecular weight around ~21 kDa, BK *β*1‐subunit protein expression should either be absent, or it would be detected as a truncated lower molecular weight protein in tissues from BK *β*1‐KO mice, if the terminator is not fully functional, and the immunogenic site for each antibody was expressed. However, all tested antibodies either failed to detect proteins at the appropriate molecular weight in tissues from WT mice, or the antibodies detected identical protein bands in tissues from WT and BK *β*1‐KO mice using the method described above. These results suggest that the proteins detected by these antibodies are not specific for the BK *β*1‐subunit; even though some of the bands could be blocked by preincubation of the primary antibodies with its competing peptide.

We tested six antibodies not only in BK *β*1‐subunit‐specific SM tissues (arteries and colons) but also tested in kidneys, a BK *β*1‐subunit enriched non‐SM tissue (France et al. 2012). Results from all tissues indicate that none of the antibodies can reliably detect the BK *β*1‐subunit. We confirmed protein loading and transfer performance on these blots by detection of *β*‐actin on the same membrane. We also reliably detected the BK *α*‐subunit protein in these tissues by western blot. Finally, we confirmed BK *β*1‐subunit gene deletion in the BK *β*1‐KO mice using real‐time RT‐PCR on the same tissue samples used for western blot analysis. In addition, published works from our group and others have demonstrated the functional deficiencies of BK channel in tissues from BK *β*1‐KO mice (Brenner et al. 2000; Petkov et al. 2001; Grimm et al. 2007, 2009; Semenov et al. 2011; Xu et al. 2011, 2012, 2014; France et al. 2012; Evseev et al. 2013; Lynch et al. 2013; Wan et al. 2013), these studies support the absence of *β*1‐subunit in BK *β*1‐KO mice.

It may be possible that loss of epitope(s) could account for the lack of band detection and lack of specificity when comparing the tissues from WT and BK *β*1‐KO mice. For this very reason, we tested several antibodies which are claimed to be specific for the BK *β*1‐subunit. Because multiple epitopes can be identified by each polyclonal antibody, this problem is less likely in our studies. Two groups have previously reported western blot data using the sc‐14749 antibody in tissues from BK *β*1‐KO mice. Loot et al. (Loot et al. 2012) studied BK *β*1‐subunit expression in cultured pulmonary arterial SMCs from WT and BK *β*1‐KO mice. However, the mouse strain used to generate the BK *β*1‐KO mice used in their study differs from ours (Brenner et al. 2000; Plüger et al. 2000). Those BK *β*1‐KO mice were generated on B6CBAF1/J (female) and deleted exon 1 of BK *β*1‐subunit gene (Plüger et al. 2000). Grimm et al. (Grimm et al. 2007, 2009) reported a ~28 kDa protein band in extracts from kidney cortex and adrenal glands from WT mice, which was absent in tissues from BK *β*1‐KO mice (Grimm et al. 2007, 2009). The BK *β*1‐KO mice used in this study were generated from the same mouse strain used in our studies. When we used the sc‐14749 antibody we failed to detect a protein band at the predicted molecular weight (~28 kDa) in protein extracts from MA or colons from WT mice, but we did detect a 28‐kDa protein band in kidney tissues from both WT and BK *β*1‐KO mice. Although the protein band is at the manufacturer's suggested molecular weight of BK *β*1‐subunit protein, we cannot confirm that this band is the BK *β*1‐subunit protein. Since all tested antibodies are polyclonal antibodies, the different results may be caused by this polyclonal antibody being produced from a different harvest (bleeding or animal) and hence a different lot. Unfortunately, we were unable to track and identify the lot number in previously published work. This could explain why our result differs from theirs. This does draw attention to the importance of verifying that an antibody is recognizing the target protein and studies in tissues from knockout mice provide this important verification. In addition, for those western blots where we detect no band at the appropriate molecular weight, we do not believe this is secondary to proteolysis as SDS is also a potent inhibitor of enzymes and we also used several protease inhibitors in the lysis buffer including NaF, activated Na_3_VO_4_, and a standard protease inhibitor cocktail. Similar to our methods, all previously published literatures used an ionic detergent (SDS) and denaturing conditions for both the sample preparation and PAGE in western blot in detecting of BK *β*1‐subunit. Furthermore, the concentration of SDS seems appropriate (1–2%) as well and we are seemingly able to identify the BK channel *α*‐subunit by western blot. Taken together, our results underscore the importance of providing confirmation of a particular immunoreactive protein on a western blot, which is one of the major points of our manuscript.

Studies have been done using custom‐made antibodies (Lu et al. 2012; Yi et al. 2014) or an antibody produced by the Merck pharmaceutical company (Matharoo‐Ball et al. 2003; Yang et al. 2009; Howitt et al. 2011). The antibody from Merck is no longer available and we were unable to test this antibody. In addition, the cell lines transfected with BK *β*1‐subunit could be a useful positive control to test the specificity of antibodies, but we are unable to establish or access this cell line.

Our data indicate that several commercially available BK *β*1‐subunit antibodies may lack specificity and sensitivity in our western blot protocols. Our studies reemphasize that proper controls must be employed when dealing with protein expression in the western blots, the issues have been recognized by others already (Saper 2005). Our study also shows that identifying bands at manufacturer's recommended molecular weight and being able to remove these bands using blocking peptide is not sufficient to conclude that the target protein has been detected. The appropriate negative control including studies in tissues from knock out animals is crucial to test the specificity of an antibody. This is especially important in the studies related to human diseases in which changes in expression of BK *β*1‐subunit expression might have an important role.

## Acknowledgments

We thank Dr. Robert Brenner (University of Texas Health Science Center at San Antonio, TX) for the gift of BK *β*1‐KO mice.

## Conflict of Interest

None declared.

## References

[b1] Ahn Y. T. Kim Y. M. Adams E. Lyu S. C. Alvira C. M. Cornfield D. N. 2012 Hypoxia‐inducible factor‐1*α* regulates KCNMB1 expression in human pulmonary artery smooth muscle cells. Am. J. Physiol. Lung Cell. Mol. Physiol.; 302:L352-L359. 22114151 10.1152/ajplung.00302.2011 PMC3289270

[b2] Albarwani S. Al‐Siyabi S. Baomar H. Hassan M. O. 2010 Exercise training attenuates ageing‐induced BKCa channel downregulation in rat coronary arteries. Exp. Physiol.; 95:746-755. 20139169 10.1113/expphysiol.2009.051250

[b3] Braun M. Ramracheya R. Bengtsson M. Zhang Q. Karanauskaite J. Partridge C. 2008 Voltage‐gated ion channels in human pancreatic beta‐cells: electrophysiological characterization and role in insulin secretion. Diabetes; 57:1618-1628. 18390794 10.2337/db07-0991

[b4] Brenner R. Peréz G. J. Bonev A. D. Eckman D. M. Kosek J. C. Wiler S. W. 2000 Vasoregulation by the beta1 subunit of the calcium‐activated potassium channel. Nature; 407:870-876. 11057658 10.1038/35038011

[b5] Chang T. Wu L. Wang R. 2006 Altered expression of BK channel beta1 subunit in vascular tissues from spontaneously hypertensive rats. Am. J. Hypertens.; 19:678-685. 16814121 10.1016/j.amjhyper.2006.01.014

[b6] Evanson K. Bannister J. P. Leo M. D. Jaggar J. H. 2014 LRRC26 is a functional BK channel auxiliary *γ* subunit in arterial smooth muscle cells. Circ. Res.; 115:423-431. 24906643 10.1161/CIRCRESAHA.115.303407 PMC4119551

[b7] Evseev A. I. Semenov I. Archer C. R. Medina J. L. Dube P. H. Shapiro M. S. 2013 Functional effects of KCNQ K(+) channels in airway smooth muscle. Front. Physiol.; 4:277 24109455 10.3389/fphys.2013.00277 PMC3791379

[b8] France M. Bhattarai Y. Galligan J. J. Xu H. 2012 Impaired propulsive motility in the distal but not proximal colon of BK channel *β*1‐subunit knockout mice. Neurogastroenterol. Motil.; 24:e450-e459. 22830588 10.1111/j.1365-2982.2012.01981.x PMC3425659

[b9] Grimm P. R. Foutz R. M. Brenner R. Sansom S. C. 2007 Identification and localization of BK‐beta subunits in the distal nephron of the mouse kidney. Am. J. Physiol. Renal. Physiol.; 293:F350-F359. 17459953 10.1152/ajprenal.00018.2007

[b10] Grimm P. R. Irsik D. L. Settles D. C. Holtzclaw J. D. Sansom S. C. 2009 Hypertension of Kcnmb1‐/‐ is linked to deficient K secretion and aldosteronism. Proc. Natl Acad. Sci. USA; 106:11800-11805. 19556540 10.1073/pnas.0904635106 PMC2701967

[b11] Howitt L. Sandow S. L. Grayson T. H. Ellis Z. E. Morris M. J. Murphy T. V. 2011 Differential effects of diet‐induced obesity on BKCa {beta}1‐subunit expression and function in rat skeletal muscle arterioles and small cerebral arteries. Am. J. Physiol. Heart Circ. Physiol.; 301:H29-H40. 21536854 10.1152/ajpheart.00134.2011

[b12] Hu X. Q. Xiao D. Zhu R. Huang X. Yang S. Wilson S. M. 2012 Chronic hypoxia suppresses pregnancy‐induced upregulation of large‐conductance Ca2+‐activated K+ channel activity in uterine arteries. Hypertension; 60:214-222. 22665123 10.1161/HYPERTENSIONAHA.112.196097 PMC3562497

[b13] Kunduri S. Dick G. Nayeem M. Mustafa S. 2013 Adenosine A1 receptor signaling inhibits BK channels through a PKC*α*‐dependent mechanism in mouse aortic smooth muscle. Physiol. Rep.; 1:e0037 10.1002/phy2.37 PMC3747964 23977428

[b14] Leo M. D. Bannister J. P. Narayanan D. Nair A. Grubbs J. E. Gabrick K. S. 2014 Dynamic regulation of *β*1 subunit trafficking controls vascular contractility. Proc. Natl Acad. Sci. USA; 111:2361-2366. 24464482 10.1073/pnas.1317527111 PMC3926029

[b15] Loot A. E. Moneke I. Keserü B. Oelze M. Syzonenko T. Daiber A. 2012 11,12‐EET stimulates the association of BK channel *α* and *β*(1) subunits in mitochondria to induce pulmonary vasoconstriction. PLoS ONE; 7:e46065 23029390 10.1371/journal.pone.0046065 PMC3454360

[b16] Lu T. Chai Q. Yu L. d'Uscio L. V. Katusic Z. S. He T. 2012 Reactive oxygen species signaling facilitates FOXO‐3a/FBXO‐dependent vascular BK channel β1 subunit degradation in diabetic mice. Diabetes; 61:1860-1868. 22586590 10.2337/db11-1658 PMC3379647

[b17] Lynch F. M. Withers S. B. Yao Z. Werner M. E. Edwards G. Weston A. H. 2013 Perivascular adipose tissue‐derived adiponectin activates BK(Ca) channels to induce anticontractile responses. Am. J. Physiol. Heart Circ. Physiol.; 304:H786-H795. 23292715 10.1152/ajpheart.00697.2012 PMC3602769

[b18] Matharoo‐Ball B. Ashford M. L. Arulkumaran S. Khan R. N. 2003 Down‐regulation of the alpha‐ and beta‐subunits of the calcium‐activated potassium channel in human myometrium with parturition. Biol. Reprod.; 68:2135-2141. 12606455 10.1095/biolreprod.102.010454

[b19] Meera P. Wallner M. Toro L. 2000 A neuronal beta subunit (KCNMB4) makes the large conductance, voltage‐ and Ca2+‐activated K+ channel resistant to charybdotoxin and iberiotoxin. Proc. Natl Acad. Sci. USA; 97:5562-5567. 10792058 10.1073/pnas.100118597 PMC25868

[b20] Nelson M. T. Cheng H. Rubart M. Santana L. F. Bonev A. D. Knot H. J. 1995 Relaxation of arterial smooth muscle by calcium sparks. Science; 270:633-637. 7570021 10.1126/science.270.5236.633

[b21] Nystoriak M. A. Nieves‐Cintrón M. Nygren P. J. Hinke S. A. Nichols C. B. Chen C. Y. 2014 AKAP150 contributes to enhanced vascular tone by facilitating large‐conductance Ca2+‐activated K+ channel remodeling in hyperglycemia and diabetes mellitus. Circ. Res.; 114:607-615. 24323672 10.1161/CIRCRESAHA.114.302168 PMC3954117

[b22] Pabbidi M. R. Mazur O. Fan F. Farley J. M. Gebremedhin D. Harder D. R. 2014 Enhanced large conductance K+ channel activity contributes to the impaired myogenic response in the cerebral vasculature of Fawn Hooded Hypertensive rats. Am. J. Physiol. Heart Circ. Physiol.; 306:H989-H1000. 24464756 10.1152/ajpheart.00636.2013 PMC3962634

[b23] Petkov G. V. Bonev A. D. Heppner T. J. Brenner R. Aldrich R. W. Nelson M. T. 2001 Beta1‐subunit of the Ca2+‐activated K+ channel regulates contractile activity of mouse urinary bladder smooth muscle. J. Physiol.; 537:443-452. 11731577 10.1111/j.1469-7793.2001.00443.x PMC2278973

[b24] Plüger S. Faulhaber J. Fürstenau M. Löhn M. Waldschütz R. Gollasch M. 2000 Mice with disrupted BK channel beta1 subunit gene feature abnormal Ca(2+) spark/STOC coupling and elevated blood pressure. Circ. Res.; 87:E53-E60. 11090555 10.1161/01.res.87.11.e53

[b25] Saper C. B. 2005 An open letter to our readers on the use of antibodies. J. Comp. Neurol.; 493:477-478. 16304632 10.1002/cne.20839

[b26] Semenov I. Wang B. Herlihy J. T. Brenner R. 2011 BK channel *β*1 subunits regulate airway contraction secondary to M2 muscarinic acetylcholine receptor mediated depolarization. J. Physiol.; 589:1803-1817. 21300746 10.1113/jphysiol.2010.204347 PMC3099031

[b27] Shi L. Liu B. Li N. Xue Z. Liu X. 2013a Aerobic exercise increases BK(Ca) channel contribution to regulation of mesenteric arterial tone by upregulating *β*1‐subunit. Exp. Physiol.; 98:326-336. 22660813 10.1113/expphysiol.2012.066225

[b28] Shi L. Liu X. Li N. Liu B. Liu Y. 2013b Aging decreases the contribution of MaxiK channel in regulating vascular tone in mesenteric artery by unparallel downregulation of *α*‐ and *β*1‐subunit expression. Mech. Ageing Dev.; 134:416-425. 24051206 10.1016/j.mad.2013.09.001

[b29] Wan E. Kushner J. S. Zakharov S. Nui X. W. Chudasama N. Kelly C. 2013 Reduced vascular smooth muscle BK channel current underlies heart failure‐induced vasoconstriction in mice. FASEB J.; 27:1859-1867. 23325318 10.1096/fj.12-223511 PMC3633822

[b30] Xia X. M. Ding J. P. Lingle C. J. 1999 Molecular basis for the inactivation of Ca2+‐ and voltage‐dependent BK channels in adrenal chromaffin cells and rat insulinoma tumor cells. J. Neurosci.; 19:5255-5264. 10377337 10.1523/JNEUROSCI.19-13-05255.1999 PMC6782330

[b31] Xie M. J. Ma Y. G. Gao F. Bai Y. G. Cheng J. H. Chang Y. M. 2010 Activation of BKCa channel is associated with increased apoptosis of cerebrovascular smooth muscle cells in simulated microgravity rats. Am. J. Physiol. Cell Physiol.; 298:C1489-C1500. 20457834 10.1152/ajpcell.00474.2009

[b32] Xu H. Garver H. Galligan J. J. Fink G. D. 2011 Large‐conductance Ca2+‐activated K+ channel beta1‐subunit knockout mice are not hypertensive. Am. J. Physiol. Heart Circ. Physiol.; 300:H476-H485. 21131476 10.1152/ajpheart.00975.2010 PMC3044058

[b33] Xu H. Wang Y. Garver H. Galligan J. J. Fink G. D. 2012 Vascular BK channel deficiency exacerbates organ damage and mortality in endotoxemic mice. J. Cardiovasc. Pharmacol.; 59:207-214. 21997262 10.1097/FJC.0b013e31823b493b PMC4327844

[b34] Xu H. Garver H. Fernandes R. Galligan J. J. Fink G. D. 2014 Altered L‐type Ca2+ channel activity contributes to exacerbated hypoperfusion and mortality in smooth muscle cell BK channel deficient septic mice. Am. J. Physiol. Regul. Integr. Comp. Physiol.; 307:R138-R148. 24829499 10.1152/ajpregu.00117.2014 PMC4101616

[b35] Yang Y. Murphy T. V. Ella S. R. Grayson T. H. Haddock R. Hwang Y. T. 2009 Heterogeneity in function of small artery smooth muscle BKCa: involvement of the beta1‐subunit. J. Physiol.; 587:3025-3044. 19359368 10.1113/jphysiol.2009.169920 PMC2718259

[b36] Yang Y. Li P. Y. Cheng J. Mao L. Wen J. Tan X. Q. 2013 Function of BKCa channels is reduced in human vascular smooth muscle cells from Han Chinese patients with hypertension. Hypertension; 61:519-525. 23232643 10.1161/HYPERTENSIONAHA.111.00211

[b37] Yi F. Wang H. Chai Q. Wang X. Shen W. K. Willis M. S. 2014 Regulation of Large Conductance Ca2+‐activated K+ (BK) Channel *β*1 Subunit Expression by Muscle RING Finger Protein 1 in Diabetic Vessels. J. Biol. Chem.; 289:10853-10864. 24570002 10.1074/jbc.M113.520940 PMC4036198

[b38] Zhang D. M. He T. Katusic Z. S. Lee H. C. Lu T. 2010 Muscle‐specific f‐box only proteins facilitate bk channel *β*(1) subunit downregulation in vascular smooth muscle cells of diabetes mellitus. Circ. Res.; 107:1454-1459. 20966391 10.1161/CIRCRESAHA.110.228361 PMC3076051

[b39] Zheng Y. M. Park S. W. Stokes L. Tang Q. Xiao J. H. Wang Y. X. 2013 Distinct activity of BK channel *β*1‐subunit in cerebral and pulmonary artery smooth muscle cells. Am. J. Physiol. Cell Physiol.; 304:C780-C789. 23426969 10.1152/ajpcell.00006.2012 PMC3625806

